# Meeting in the margin: can participatory research address the root causes of Indigenous mental health problems?

**DOI:** 10.1177/11771801241251456

**Published:** 2024-05-21

**Authors:** Georgia Vrakas, Arlene Laliberté (Timiskaming First Nation)

**Affiliations:** 1 Center for Research and Intervention on Suicide and End of Life Practices, Canada; 2 Université du Québec à Trois-Rivières, Canada

**Keywords:** anti-oppressive research, empowerment, Indigenous mental health, oppression, participatory research

## Abstract

Although Indigenous Peoples represent 5% of the population in Canada, they present higher rates of psychosocial problems including mental health issues and suicide than their non-Indigenous counterparts. They are also over-represented in the youth protection and prison systems. This must be understood within the specific context of the oppression of Indigenous Peoples in Canada through colonization and colonialist policies. To improve Indigenous mental health and wellbeing, the oppression underlying it must be addressed. The objective of this article is to illustrate how we, as mental health researchers, can contribute to this process. Based on Collins’ matrix of domination, and bell hooks’s space of resistance, an analysis of community-based participatory research and its impacts on helping Indigenous People overcome oppression is offered. Limits of participatory research’s contributions to social change are presented. Anti-oppressive participatory research is offered as a promising alternative.

## Introduction

Three distinct groups comprise Indigenous Peoples in Canada: First Nations who are neither Métis (Indigenous People who are descendants of communities originating from the Red River Valley in what is now the province of Manitoba) nor Inuit (the Indigenous People of the Artic) (Government of Canada, 2021; Vowel, 2016). Each group has their own unique histories, cultures, realities and experiences (Vowel, 2016).

There are 1.8 million Indigenous Peoples in Canada according to the 2021 Census, representing 5% of the population (Statistics Canada, 2023). The growth rate of Indigenous population, however, is twice that of the non-Indigenous population in Canada. Furthermore, the Indigenous population is 8 years younger than their non-Indigenous counterparts. Their mean age is 33.6 versus 41.8 years for the non-Indigenous population (Statistics Canada, 2023). Recent surveys reveal Indigenous disadvantage across many socio-economic and psychosocial domains, affecting among other things their mental health and wellness. These high rates of psychosocial problems should be understood within Canada’s colonization of Indigenous Peoples and its effects (Statistics Canada, 2019), as well as its colonialist assimilationist laws and practices which continue to have a deleterious impact on Indigenous lives today (Manuel & Derrickson, 2015, 2017).

Indeed, in the past few years, government-mandated commissions have investigated the residential school system (Commission de la vérité et de la réconciliation, 2015), the phenomenon of missing and murdered Indigenous girls and women (MMIGW) (Enquête nationale sur les femmes et les filles autochtones disparues et assassinées, 2019), systemic racism (Commission d’enquête sur les relations entre les Autochtones et certains services publics, 2019) and their effects on the lives of Indigenous Peoples. The results of these public inquiries reveal that Indigenous Peoples still suffer today from the traumatic effects of the residential school system. Indeed, Indigenous women and girls are far more at risk of being killed or disappearing than their non-Indigenous peers, and Indigenous men and women face discrimination from the police, the justice and correctional system, the health and social services system as well as youth protection services, all of which are publicly funded and publicly run. The Truth and Reconciliation Commission in 2015 concluded that Indigenous Peoples had suffered a cultural genocide at the hands of Canada. The MMIWG went one step further and stated that Canada had committed genocide as defined by international law.

As researchers aiming to work with Indigenous groups to help them address their mental health problems, we cannot ignore the social and economic inequalities they face rooted in the particular Canadian colonial context. Based on the existing literature (Institut canadien d’information sur la santé, 2009; Public Health Agency of Canada, 2022; World Health Organization, 2018), we define mental health as a positive and holistic concept that emphasizes cultural, community, family and personal resources as well as spiritual, mental and physical capacities. Indigenous communities adhere to this definition of mental health in which each individual is able to achieve their full potential as a human being, thereby bringing about the total wellbeing of their community, and it includes the cyclical concept of life-death-life (Kirmayer et al., 2000). This life-death-life cycle is one of inter-relatedness. In Nature, when things die, they nurture new life. Human beings are also part of that cycle. They learn from their ancestors to teach their children. They carry their ancestors with them, and they make decisions with their children and future generations in mind. The cycle is both physical as in the death of the body, and spiritual that is the connection to the spirit world.

## Social inequalities and psychosocial problems

Examples of social inequalities affecting Indigenous Peoples are numerous. In 2021, 17.1% of Indigenous Peoples in Canada were living in overcrowded housing, meaning, more than one person per room (Statistics Canada, 2022a). In comparison, 9.4% of non-Indigenous Canadians lived in overcrowded housing (Statistics Canada, 2022a). Furthermore, 47% of First Nations, 25% of Inuit and 22% of Métis children live in poverty versus 12% of non-Indigenous children (Beedie et al., 2019).

Indigenous People in Canada experience higher rates of psychosocial problems than their non-Indigenous counterparts. According to the most recent available data, in 2023, 26.1% of Indigenous People aged 15 years and over perceived their mental health as being fair or poor versus 19.4% of their non-Indigenous counterparts (Statistics Canada, 2024). Between 2011 and 2016, the suicide rate in First Nations was three times higher than that of the general Canadian population. Among Inuit, this rate was nine times superior to that of the general population (Statistics Canada, 2019).

In 2019, the General Social Survey on Canadians’ Safety concerning people aged 15 years and older indicated that 8.4% of Indigenous People reported having been victims of violence compared to 4.2% of the non-Indigenous population (Statistics Canada, 2022a). Moreover, 41% of Indigenous respondents indicated having been victims of sexual or physical assault before the age of 15, versus 25% for non-Indigenous Canadians (Statistics Canada, 2022b).

These rates of childhood mistreatment are reflected by the high present-day numbers of Indigenous children placed in youth protection, also referred to as the new residential school system by some (Commission de la vérité et de la réconciliation, 2015). Among all the children in the Canadian foster-care system under 14 years of age, 53.8% were Indigenous even though they only represent 7.7% of the children in Canada of the same age group (Government of Canada, 2023).

This alarming portrait of Indigenous mental wellness problems in Canada demonstrates the necessity to address the underlying causes of these issues.

## Mental health and oppression

Research has revealed that oppression through colonization is considered to be the root cause of high levels of mental health problems and suicide in Indigenous communities (Kirmayer et al., 2003; Wexler, 2006; Wexler & Gone, 2012). Indigenous Peoples perceive these problems as having social and political origins (Kassam, 2006; Kirmayer et al., 1997, 2003). Considering that Indigenous People’s mental health and wellness problems are anchored in past and ongoing oppression resulting from Canadian colonization and colonialist policies, we contend that in order to foster wellbeing, this oppression must be addressed and ultimately overcome. Hence, the main objective of this article is to examine how we, as researchers, can address this oppression through our mental health research with Indigenous Peoples.

First, however, it is important to note our specific positions in this article. We are two community psychologists with backgrounds in suicidology, specialized in research with Indigenous Peoples with a profound understanding of the historical and structural factors creating the contextual challenges to wellbeing faced on a daily basis by Indigenous Peoples. The second author is an Algonquin (one of the ten First Nations in Quebec and Ontario, Canada) woman (Government of Canada, 2022). Her doctoral research focused on suicide in First Nations followed by 5 years of postdoctoral work on Indigenous mental promotion and prevention. She is now a clinical psychologist and consultant with First Nations communities in Quebec, including her own, since 2016. The first author is a member of a minority ethnic group, a second-generation Greek immigrant and a university professor having worked with Inuit communities in Nunavik, Quebec, Canada, for over 10 years first as a mental health researcher then as a pedagogical counselor and a consulting psychologist. We are both aware of our unique positions, both of privilege as researchers and of outsiders; that is, both of our positions of privilege place us outside, albeit in different ways, from the Indigenous communities we work with.

## Addressing oppression through empowerment-based research

For Prillenltensky and Gonick (1996), there are two dimensions to oppression: the political one in which one group puts barriers into place in order to prevent another group from participating fully in society, defining their identity and accessing resources; and the psychological one, rooted in the political dimension, characterized by the internalization of feelings of inferiority by the dominated groups.

Patricia Collins offers a more complex theory in which race, gender, class, sexuality, nation, age and ethnicity are forms of oppression (Collins, 2009). Although hers is a feminist theory based on the standpoint of Black African American women, we believe it can provide a useful lens to analyze Indigenous oppression and develop ways to challenge this oppression. According to Collins, the complex relationship between the oppressor and the oppressed must be understood within a matrix of domination, which is the organization of power relations in a given society characterized by specific intersecting systems of oppression, such as race, ethnicity, gender, social class and a distinct organization of its domains of power. The first domain is structural, based on the organization of social institutions in order to reproduce and maintain oppression, for example, in Canada: the residential school system; government policies put into place to keep Indigenous Peoples in subjugated states such as the Indian Act 1876 (Ministère de la Justice du Canada, 2019). The second is disciplinary which is exercised through, among others, power relations via bureaucracy, surveillance and different rules for different groups. Examples of this are the higher rates of Indigenous children placed in youth protection versus their non-Indigenous counterparts, as well as systemic racism (Office de consultation publique de Montréal, 2019). The hegemonic domain encompasses ideology, culture and consciousness, the narratives created and maintained by the dominant group. The erasure of Indigenous Peoples in the history of Canada presented in school curricula (Truth and Reconciliation Commission of Canada, 2015) and the negative portrayal of Indigenous People in the media (Harding, 2006; King, 2017; McCue, 2014) are examples of this. Finally, we have the interpersonal domain which includes the everyday ways people treat each other and themselves, including internalized oppression (Collins, 2009).

Collins’s matrix of domination raises two questions. First, how do oppressed and disempowered groups gain power in contexts where structures, discipline, ideology and everyday practices maintain them in their subjugated states? Second, how can we, as mental health researchers, participate in this process of empowerment and help promote wellness? These two questions are crucial considering that research and academia have a role in maintaining oppression through both the structural and hegemonic domains of power by (1) the choices we make regarding our objects of study (Sandler, 2007), (2) relegating Indigenous epistemologies to the category of myth (Battiste, 2005; Qwul’sih’yah’maht, 2015; Watt-Cloutier, 2016; Williams & Mumtaz, 2008) and (3) more often than not, taking over the voices of the oppressed groups and speaking for them (hooks, 1989) in our scientifically approved language in order to have this knowledge considered as being valid by our scientific and academic peers (Collins, 2009).

Considering that knowledge is necessary for resisting oppression, for fostering empowerment and for achieving wellness, how do researchers participate in the production of knowledge to address these roots of mental health issues and ultimately truly promote wellness as it is understood and experienced by Indigenous People?

Participatory research seems to be a way forward because of its emphasis on empowerment (Laliberté et al., 2009; Rappaport, 1981) and putting forth the voices of the communities and people involved as partners in the process not as objects of inquiry (Minker & Wallerstein, 2003). It offers, as we shall see, a way to challenge the hegemonic, the interpersonal domains and even the structural domains of power (Collins, 2009).

Community-based participatory research (CBPR) (Minkler & Wallerstein, 2003) is an approach in which institutional researchers and community organizations are partners, making decisions together and equitably contributing throughout the entire research process. CBPR involves active participation and cooperation between researchers and communities, permitting individuals, organizations and communities to increase control over their lives and situations through the co-construction of contextualized knowledge and the development of scientifically based, adapted local solutions and building capacity to address present and future health issues (Macaulay et al., 1999). Furthermore, it allows the community research partners to appropriate the research process and the results as the study evolves. It ensures that knowledge use is integrated from the inception of the project, the data collection, the analysis and the interpretation to the community-wide dissemination of the results. Participating in the research and using the knowledge it generates is itself an empowerment process.

Empowerment can be defined as “an active, participatory process through which the individuals themselves gain greater control, efficacy and social justice” (Laliberté et al., 2009, p. S65). The concept of empowerment acknowledges that people have the competencies to meet their own needs (Rappaport, 1981). It is congruent with the worldview of many Indigenous Peoples in which working together and a sense of being part of a whole are at the center of a community-minded mentality. Laliberté and colleagues (2009) advocate for and present examples of ways of thinking and doing health promotion that begin with empowerment to help people gain a greater level of control over their lives and circumstances. Various researchers have used participatory approaches in projects with Indigenous People on a diversity of mental health-related topics. Here are some examples of how this type of research can challenge the hegemonic, interpersonal and structural domains of power or at least bring them to the forefront.

Wexler’s (2006, 2009; Wexler & Gone, 2012) research on Inupiat (the Inuit of the northern and northwestern Artic region of Alaska, USA) youth suicide in Alaska highlights the importance of developing knowledge to better understand this issue as it is lived, experienced and perceived by Indigenous Peoples themselves rather than what the Western hegemonic view assumes it to be. Results of Wexler’s research (Wexler, 2006, 2009; Wexler & Gone, 2012) show that colonization is indeed perceived as having led to problems such as suicide. However, the Inupiat youth blame themselves for the present problems, which can be seen as a form of internalized oppression within Collins’ (2009) interpersonal domain. They do not see a future for themselves because they see their choices as being predetermined by the examples set by the adults in their community, and they blame them for their failures (Wexler, 2009). The more subtle forms of ongoing modern colonization and oppression within the structural domain and its impacts on the current problems faced by the people in the community are not perceived nor understood as being a cause of these issues by the Inupiat youth.

Wexler suggests that these perceptions can be changed if the youth better understood the ongoing negative impacts of colonization on themselves, their communities and cultural discontinuity instead of blaming themselves for problems they face such as suicide. Through this understanding, they can develop their own meanings of wellness and perspectives for a future rather than have them imposed upon them by the dominant discourse conveyed by the oppressor.

Key elements in this participatory research process helped the Inupiat develop knowledge on suicide and actions to prevent suicide in their communities. First, Wexler conducted this project on Inupiat youth suicide while being the suicide-prevention coordinator for the region during the study. Second, interviews, focus groups with youth and surveys were shared and discussed with the community members in order to develop suicide-prevention and wellness plans for the region’s communities. Third, the Regional Suicide Prevention Task Force helped facilitate the research process. Finally, Wexler responded to the needs of the community by providing suicide-prevention workshops and services for the youth of the region. Thus, what emerges from this participatory research is that being in the community, working with the community and meeting the needs of the community are crucial elements for enacting change.

Riecken and colleagues’ (2006) project on Traditional Pathways to Health is another example of a participatory study involving Indigenous Peoples. In this project, First Nations’ high school students were co-researchers. They had to choose a health or wellness topic, for example, drug and alcohol use, suicide, protective effects of culture and healthy lifestyles, that interested them and proceeded in planning and conducting the research. They each became in effect researchers of their own projects aiming to answer the research question of their own choosing. They researched the question through interviews and document gathering. They produced videos on their topic that they then presented to the community, thus disseminating research results. Some students’ videos focused on problems they faced, but most focused on positive aspects of their communities and their cultures such as traditional foods and healing circles. By creating new and locally embedded participatory research processes and knowledge on Indigenous wellness, which put culture at the forefront and validated their worldviews on the subject, they resisted the dominant negative worldview (Riecken, Scott, et al., 2006), thus challenging the hegemonic domain of power. Furthermore, they challenged the structural domain of power within a traditional school system as is reflected by a certain resistance manifested by some school personnel toward the project.

In order to develop participatory research that considers Indigenous worldviews, several key steps must be followed. Constructing relevant partnerships is a crucial first step. They bring together university researchers and community organizations and members to work collaboratively on the research process (Minkler & Wallerstein, 2003). In this way, structural power is shared. One avenue for creating and sustaining these partnerships is the application of the Ownership, Control, Access and Possession (OCAP^®^) principles regarding research with First Nations (First Nations Information Governance Centre, 2007, 2023). These principles are a way to ensure First Nations’ self-determination in the research domain. In the past, research with Indigenous Peoples often was done on them not with them. They were not consulted by the researchers and were considered as subjects. Furthermore, research has been used to oppress and stigmatize them. These past negative experiences have led First Nations to want to engage in research differently, in that they wish to control all stages of a project involving them. They want research that responds to their needs and that is conducted in an ethical manner. The OCAP^®^ principles are a way to ensure that First Nations decide what type of research to be part of, how the research will be conducted from the beginning to the end, how the collected data will be used, where these data will be stored, who will have access to it and to whom it will belong.

It must be noted that the three major Canadian federal funding agencies have clear requirements for researchers wishing to engage in research with First Nations, Inuit and Métis. To obtain funds from these agencies, researchers must follow these requirements. Some examples of these are: community engagement in the research, respect for Indigenous authorities, engagement with the Indigenous organizations, implementation of research agreements, participatory research. Indeed, Chapter 9 of the *Tri-Council Policy Statement: Ethical Conduct for Research Involving Humans* (Secretariat on Responsible Conduct of Research, 2018) is exclusively dedicated to research involving the First Nations, Inuit and Métis Peoples of Canada and outlines all the requirements that must be respected by researchers working with Indigenous Peoples.

Participatory research can be a vehicle for decolonizing research, so that Indigenous views and voices lead the process of research development from formulating objectives, proposing methods, to results utilization. This power-sharing ensures the relevance of the research for the communities involved (Bartlett et al., 2007). Participatory research which respects the OCAP^®^ principles makes it that those of us who usually have power in research and academia let go of, at least part of, that power. This is necessary for the partnerships to be authentic and for power to be truly shared (McIntosh, 1989; Nelson & Prilleltensky, 2005). Indeed, sharing power means demonstrating respect toward our partners, their experiences, their expertise, their ways of knowing, being and doing.

Developing authentic relationships between researchers and communities is paramount when conducting research with Indigenous People. It is achieved through, among others, community consent, sharing of power regarding the research process, collaborative interpretation and dissemination of the results and doing research that is relevant and brings benefits to the community (Bull, 2010).

Nelson and colleagues (2001) go one step further and propose that when working with oppressed groups, we develop value-based partnerships as a way to strive for solidarity with them. What this means is that the partnership should exist *for* the benefit of the oppressed because of the power imbalance that generally exists between professionals and the oppressed groups within the structural, hegemonic and interpersonal domains of power. If our research aims to bring about social change, solidarity is necessary for transformation of the oppressive situation (Freire, 2007).

## Meeting in the margin: Indigenous resistance

Inasmuch as the partnership between research and community is essential in participatory research, it is important that researchers respect and recognize the space, that gap which symbolizes the relationship of inequality between colonizer and colonized, which Jones and Jenkins (2008) call the “hyphen” (p. 471). For them, we cannot act as though that gap has been erased once a research partnership is created; doing so, would be engaging in recolonization. Jones and Jenkins propose that researchers learn *from* Indigenous People and hear them speak in their own voices instead of learning *about* them, through the voice of the researcher (Jones & Jenkins, 2008). hooks (1989), a Black feminist theorist, calls this “meeting in the margin” (p. 19). For her, speaking about the other further oppresses. The margin is thus a space of resistance. It is the crucial space where people who are oppressed resist the oppressor by creating counter-hegemonic ways of being and knowing (hooks, 1989). Collins (2009) speaks of lived experience as being the basis for knowledge to be considered valid and meaningful. It must come from the oppressed themselves, their lived experiences. This knowledge is necessary for the transformation of the relationship between oppressed-oppressor (Allen, 2002). hooks (1989) calls for an erasure of the category colonizer-colonized by standing in solidarity in the margin, meaning that the oppressed speak for themselves through their own voices—not appropriated by the colonizer. Their ways of knowing and being are put forth as ways of resisting oppression.

Indigenous narratives of resistance, of ways of knowing and seeing the world and being have been found to promote resilience (Kirmayer et al., 2011) which is associated to wellbeing (Mitchell & Ezcurra, 2017). Creating new narratives by reviewing history through their eyes, by focusing, among others, on Indigenous identity, revitalization of their language, their culture and spirituality, on political activism and reconciliation promote individual and collective resilience (Kirmayer et al., 2011). For Wexler and colleagues (2009), making sense of the collective experience of adversity instead of internalizing oppression promotes resilience in Indigenous people as does having a strong cultural identity. This underscores the importance of knowledge produced by the oppressed themselves about themselves, on their ways of being and knowing, in a space where they can produce these counter-hegemonic narratives as a way to resist oppression and to foster empowerment.

Our role as researchers is to help reveal these narratives, through the use of methods that support the voices of the people we work with (Kirkpatrick, 2008; Rappaport, 1995, 2000) as we engage in CBPR, driven by the needs and the voices of our partners. What research methods can we use to change things and bring forth the knowledge created by the oppressed groups we work with?

Photovoice is one option. Taken literally, it means voice through photography. Photovoice is a method informed by Paolo Freire’s empowerment education theory (Freire, 2005), feminist theory and documentary photography (Wang & Burris, 1997). It is meant to empower people from marginalized groups, those who do not usually have a voice. Through photos, they can tell their stories about issues facing their communities in order to advocate for change. Although participatory research takes many forms, photovoice is a participatory method that has been extensively used to support Indigenous People voices and work in the margins to deconstruct colonialist views of these groups. Photovoice (Wang, 2003) is one of the ways we can use to help create these narratives (Palibroda et al., 2009). It is a qualitative visual data-gathering method where the participants take pictures to express themselves regarding issues that are important to them such as food security, community safety, mental health and drug use. They then together discuss and analyze the photos they have taken within a group setting in order to share their experiences regarding the issue at hand. This group dialogue is meant to foster their collective critical consciousness (Freire, 2007) regarding their community’s problems and generate ideas for solutions to these problems.

Ultimately, photovoice is meant to be used as a tool for social change. It is not only participatory research method but also a health-promotion tool (Wang, 1999; Wang et al., 1998). Photovoice is a method that has been adapted for use with Canadian First Nations’ communities (Wang & Burris, 1997). Visual methods have been used before with Inupiat youth to address suicide and resilience (Wexler et al., 2013; Wexler, 2006). Photovoice has been utilized to study various determinants of physical and mental health with Indigenous People, such as food security (Genius et al., 2014), eating habits (Kelly, 2017), drug prevention (Helm et al., 2015), underage pregnancy (Fuery et al., 2009) and healthy relationships (Markus, 2012). The main goals of photovoice (Wang & Burris, 1997) within CBPR are to help individuals, those who usually have less power, identify and reflect on community issues through picture-taking; promote group dialogue on these issues by discussing the pictures they have taken; influence decision and policy-makers, those who usually have more power, to include community produced solutions to community defined problems. Photovoice thus aims to help participants develop counter-hegemonic narratives about problems affecting them and their own solutions to address them, within a space that is theirs, the margin, in which we are privileged to be invited. It is their voices, not ours, that are expressed through the images they take and narrate. It is their stories that are put forth and presented to their communities and ours. Photovoice therefore can be a good example of participatory research approaches that can help deconstruct negative dominant discourses about Indigenous Peoples.

## Limits and future possibilities of participatory research

Although participatory research has shown a lot of promise as a way to develop knowledge by Indigenous Peoples, to put forth Indigenous voices, to value lived experience as valid knowledge, to meet in those spaces of resistance all of which aim to challenge the existing structures of power within the structural, hegemonic and interpersonal domains, there are limits in the role participatory research has in affecting social change.

How effective is participatory research against oppression? Here, Sandler’s (2007) point is of utmost relevance: working on increasing the power of communities versus the power of universities “is not in itself a step toward social justice; doing so does not address the fundamental problem of the existing structural injustices manifest within communities” (p. 276). As participatory researchers, our focus is on power-sharing with community organizations and members and amplifying the voices of those who do not usually have the opportunities to express themselves and be heard by those in power, in order to affect the structural and hegemonic status quo traditionally found in university-based research. It is how we work with Indigenous Peoples on Indigenous mental health, that is, through empowerment-based approaches.

Nevertheless, although empowerment is necessary, it is not sufficient on its own to overcome social injustices; social change is needed for that to happen (Allen, 2002; Collins, 2009; Nelson & Prilleltensky, 2005). Our goal is that our participatory research contributes to significant social change. Sanon and colleagues (2014) conducted a review on the use of photovoice to promote social justice, that is, the value which orients the fair and equitable allocation of resources and burdens in society (Nelson et al., 2001). They found that all the studies did raise awareness regarding social injustice, mostly at an individual level. However, only in a minority of studies (3/30) did photovoice actually have a transformative social justice impact, addressing the root causes of the problems, for example, by changing laws or improving community resources. This may be that researchers have not yet used photovoice to its full potential to promote social justice (Sanon et al., 2014). Or it may be that the support to communities required to achieve sustainable changes is beyond the scope of a single research project (Laliberté et al., 2009; Potts & Brown, 2015). Indeed, the key to sustained social justice changes may be an ongoing partnership between university-based and community partners working together in solidarity over several years to create individual and community empowerment.

As researchers, our primary task is to develop knowledge within a specific structure, the academic one which has its own set of rules. Consequently, we work mainly within the structural and hegemonic domains of dominant knowledge production. As participatory researchers, we can help counter these power structures and reveal counter-narratives created by the oppressed groups (Collins, 2009; hooks, 1989; Rappaport, 1995, 2000), thus fostering empowerment. These narratives are not necessarily new knowledge; it is perhaps new to us researchers, new to the scientific community, new to the funding agencies—as we all know, for a research project to be funded and its results published, it must focus on developing as yet undiscovered scientific knowledge. It is not new, however, to the groups we work with because it represents their views and their lived realities. According to Freire’s empowerment education theory (Freire, 2005, 2007), people have the capacity to identify their own problems and to find the solutions to meet their own needs. They have this knowledge. Our role is not to speak it in their name. Our role is two-fold: (1) fostering the development of counter-narratives by helping create opportunities to meet in the margin (hooks, 1989), where Indigenous People can express themselves, be heard and listened to; and (2) bring their voices to the forefront to ensure that these counter-hegemonic ways of being and knowing are heard. This is achieved by using both the power and privilege afforded to us by the dominant structure of academia, which permits us to engage in this type of research and by developing value-based partnerships with the communities we work with, without which we would not have the privilege to be invited into these spaces where these counter-narratives are revealed.

Nonetheless, bringing significant and lasting change to the structural domain of power which maintains the oppression of Indigenous Peoples in place is difficult. Through our own participatory research projects on Indigenous mental health (Vrakas, 2011; Vrakas & Laliberté, 2011), we worked with people on fostering empowerment at the individual and group levels using photovoice as our principal method. However, actually bringing about change at the community level, within the decision-making structures and institutions, including our own, remains a challenge.

## Anti-oppressive research

So, then what can we do? As mental health researchers working with Indigenous Peoples to improve their mental health and wellness, we need to bring change at the structural level. We may succeed in bringing about social change if we use our research to its full potential to promote, support and participate in transformative social justice. We believe that, in order to achieve this, we must adopt an anti-oppressive epistemological posture (Moosa-Mitha, 2015) within our participatory research.

Anti-oppressive research is not a specific method but a set of principles, values and way of working (Potts & Brown, 2015). It is explicitly oriented toward social justice, defying dominant ideas through both research methods and research outcomes. It is research *with* people not on people. It also demands that researchers engage in critical reflexivity. This means examining ourselves, our values, our identities, our positions of privilege as well as the ideologies rooted within our own research processes. Indeed, it requires understanding that research is not neutral, it is shaped by those in power, and that knowledge is not neutral, it is socially and politically constructed (Potts & Brown, 2015). We are not detached observers uncovering some truth. We are part of our research, and we must ask ourselves why are we studying what we are studying, why do we tend to focus on marginalized groups versus dominant ones, which evidence matters and why? The anti-oppressive researcher is committed to the people they work with and to promoting social justice.

In social sciences research, we usually tend to focus on the disadvantaged, the oppressed (to have their perspectives and voices heard), and rarely do we focus on the dominant, advantaged groups, that is, the oppressors (Allen, 2002; Potts & Brown, 2015; Stephens, 2010). As Allen (2002) points out, when reading Freire’s (2007) *Pedagogy of the Oppressed*, we identify with the oppressed not the oppressor, we being university educated and privileged, we being researchers and academics. It is hard to accept that most of us in research and academia are in the oppressor category. It is difficult for us to admit to ourselves that we are part of the dominant group.

We need to focus on those very groups, on the privileged, to understand why they, why *we* are the advantaged and how they, how *we* contribute to oppress the disadvantaged and marginalized (Potts & Brown, 2015; Stephens, 2010). In this way, by unpacking White privilege (McIntosh, 1989) and by understanding and being aware of our role as oppressors, we develop our own critical consciousness.

In our case, as researchers and psychologists, we are acutely aware of both our positions of privilege and advantage and our positions as Indigenous and ethnic minority women. These multiple perspectives and our lived experiences taint the topics we choose to study, in this case, Indigenous wellbeing and empowerment, the participatory research approaches we choose to adopt, the way we engage as partners with the communities we work with, as well as how we collaboratively collect data, interpret them and disseminate the research results. However, awareness or *conscientization* (Freire, 2005) is not enough. We must stand in solidarity with the oppressed (hooks, 1989; Nelson et al., 2001; Prilleltensky, 2008; Prilleltensky & Gonick, 1996), and crucially, we must demonstrate this solidarity by working actively and explicitly against the structures of oppression (Allen, 2002) within the community and societal levels. In working with Indigenous Peoples, our anti-oppressive research must be respectful of Indigenous ways of knowing and doing. More specifically, we must engage in Indigenous research which in itself has a decolonization objective (Kovach, 2015). This means recognizing that Indigenous research frameworks encompass both knowledge systems and methods, and thus, must be rooted in Indigenous epistemologies (Kovach, 2009).

As we demonstrated earlier, empowerment-based approaches such as participatory research are limited in bringing about social change within the structural domain of power wherein are rooted causes of the mental health problems experienced by Indigenous Peoples. Indeed, trying to foster empowerment without addressing oppression can impact our understanding of the situation and thus rendering the interventions we put into place to help people inadequate (Lord & Dufort, 1996). Adopting an anti-oppressive stance focused on bringing about social justice can however help us address and challenge oppression and foster change at the structural level. As researchers, we contend that it is essential to adopt an anti-oppressive posture in doing mental health research in partnership with Indigenous Peoples in order to help them achieve significant and durable changes in their mental health and wellbeing.

Engaging in anti-oppressive work means recognizing that it is crucial for oppressed groups to be heard, to have spaces where they can create new knowledge about themselves, their worldviews, their lived experiences, to bring about change in the hegemonic domain of power. It also means recognizing that this is not enough. To be able to change the structural domain of power, it is just as important to obtain knowledge about the oppressor (Potts & Brown, 2015). More specifically we need to turn our gaze upward toward the oppressor to understand the conditions which allow them to perpetuate the marginalization of certain groups and maintain their own positions of power and their dominance over the oppressed. This knowledge is not easily accessible as it is more difficult to research privilege and power than marginalization and oppression. Nonetheless, it is necessary to do so because knowledge and power are intimately interrelated (Macias, 2015). We need to know what allows the oppressor to continue oppressing not just for the sake of the knowledge itself but more importantly to help us gain the power to transform things at the structural level, alongside and in solidarity with the oppressed.

By focusing our research on both the oppressed and the oppressor, we can obtain knowledge and power to work against oppression in the hegemonic, interpersonal and structural domains of power and promote social justice. We can better understand and address the causes, rooted in oppression, of the mental health problems experienced by Indigenous Peoples. We can work alongside with them on fostering empowerment, on decolonization and on creating the necessary conditions for improving their wellbeing.

## Conclusion

This article aimed to illustrate how researchers can address the root causes of mental health problems in Indigenous Peoples of Canada, most specifically the oppression brought upon by past and ongoing colonization and colonialist policies and practices. We examined CBPR as a potential way forward. Through Collins’s (2009) matrix of domination, we offered an analysis of the strengths and limitations of CBPR as a tool to overcome oppression mainly within the structural and hegemonic domains. Although participatory research can be successful in developing counter-hegemonic narratives and challenging internalized oppression, it is somewhat less successful in bringing about transformative change within the structural domain of power, beyond that of academia. Adopting an anti-oppressive stance, however, can help participatory research contribute to meaningful structural change. For this to happen, certain key elements must be present, such as committing to long-lasting value-based partnerships between researchers and Indigenous Peoples, explicitly demonstrating our anti-oppressive decolonizing stance and a willingness to shift our gaze upward and inwards to face the oppressor, to face ourselves.
